# Antimicrobial activity from ticks eggs waxes

**DOI:** 10.1186/1753-6561-8-S4-P156

**Published:** 2014-10-01

**Authors:** Natalia Alduini, Marcos Silva, Marcia Franzolin, Ronaldo Mendonça, Solange Lima-Netto

**Affiliations:** 1 Instituto Butantan, São Paulo, SP, Brazil

## Introduction

Ticks lay their eggs in the environment and cover the eggs in a waxy layer to protect them from desiccation and microbial attack. This wax is produced by an organ known as Gene´s Organ. Bio prospection has shown the presence of active principles in the hemolymph of arthropods as well as in the salivary glands of ticks. Some of these are of interest for the development of new pharmacological drugs. In this study, different tick species were used to test the antimicrobial effect of the extract obtained from the wax envolving the eggs.

## Objectives

The objective of this study is to evaluate the antimicrobial effect of the waxy secretion from the eggs of the following tick species: *Amblyomma cajennense, Amblyomma aureolatum, Rhipicephalus *(*Boophilus*) *microplus *and *Rhipicephalus sanguineus*.

## Methods

The egg masses were treated with icy cold phosphate buffer (pH 6.8) to test against Influenza virus (H_1_N_1_) to determine the antiviral activity of the ticks eggs wax. MDCK cells were infected with influenza viruses, culture of MDCK cells, performed in 96 wells microplate, were treated with 2600, 1300, 650, 325, 162.5, 82, 41 and 20.5 mg/mL of the eggs wax extract 1 h before infection. After 72 h post infection cytopathic effect induced by the virus was observed, the culture medium was removed and the cells in the plate were stained with crystal violet (0.2% in 20% methanol). The egg wax was maintained in culture during the time of infection. The eggs were also treated with chloroform to obtain an extract suitable for the disc diffusion standard methodology stablished by Kirby and Bauer. The microorganisms used to test the activity were: *Candida albicans, Micrococcus luteus, Escherichia coli*, and *Staphilococcus aureus*. After incubation the plates were observed for the presence or absence of growth inhibition.

## Results

Amounts as small as 325 mg/mL of the extract were able to inhibit the replication of the virus. Besides, the sample presented very low citotoxicity on Vero cells. On the other hand, the organic extract from *A. aureolatum *and *R. sanguineus *showed an inhibition zone for the strains of *C. albicans, M.luteus, E.coli *and *S.aureus*. This result is in accordance with the antimicrobial activity reported for the wax extracted from other ticks.
